# Improving access to skilled facility-based delivery services: Women’s beliefs on facilitators and barriers to the utilisation of maternity waiting homes in rural Zambia

**DOI:** 10.1186/s12978-015-0051-6

**Published:** 2015-07-08

**Authors:** Cephas Sialubanje, Karlijn Massar, Marit S. G. van der Pijl, Elisa Maria Kirch, Davidson H. Hamer, Robert A. C. Ruiter

**Affiliations:** Ministry of Health, Monze District Medical Office, P.O. Box 660144, Monze, Zambia; Department of Work and Social Psychology, Maastricht University, P.O. Box 616, 6200MD, Maastricht, The Netherlands; Department of Global Health, Maastricht University, Faculty of Health, Medicine and Life Science, P.O. Box 616, 6200MD, Maastricht, The Netherlands; Zambia Centre for Applied Health Research and Development, P.O. Box 30910, Lusaka, Zambia; Centre for Global Health and Development Boston University, Crosstown 3rd floor, 801 Massachusetts Avenue, Boston, MA 02118 USA; Department of International Health, Boston University School of Public Health, Crosstown 3rd floor, 801 Massachusetts Avenue, Boston, MA 02118 USA

**Keywords:** Maternal health, Facility-based delivery services, Maternity waiting home, Kalomo, Zambia

## Abstract

**Background:**

Maternity waiting homes (MWHs) are aimed at improving access to facility-based skilled delivery services in rural areas. This study explored women’s experiences and beliefs concerning utilisation of MWHs in rural Zambia. Insight is needed into women’s experiences and beliefs to provide starting points for the design of public health interventions that focus on promoting access to and utilisation of MWHs and skilled birth attendance services in rural Zambia.

**Methods:**

We conducted 32 in-depth interviews with women of reproductive age (15–45 years) from nine health centre catchment areas. A total of twenty–two in-depth interviews were conducted at a health care facility with a MWH and 10 were conducted at a health care facility without MWHs. Women’s perspectives on MWHs, the decision-making process regarding the use of MWHs, and factors affecting utilisation of MWHs were explored.

**Results:**

Most women appreciated the important role MWHs play in improving access to skilled birth attendance and improving maternal health outcomes. However several factors such as women’s lack of decision-making autonomy, prevalent gender inequalities, low socioeconomic status and socio-cultural norms prevent them from utilising these services. Moreover, non availability of funds to buy the requirements for the baby and mother to use during labour at the clinic, concerns about a relative to remain at home and take care of the children and concerns about the poor state and lack of basic social and healthcare needs in the MWHs − such as adequate sleeping space, beddings, water and sanitary services, food and cooking facilities as well as failure by nurses and midwives to visit the mothers staying in the MWHs to ensure their safety prevent women from using MWHs.

**Conclusion:**

These findings highlight important targets for interventions and suggest a need to provide women with skills and resources to ensure decision-making autonomy and address the prevalent gender and cultural norms that debase their social status. Moreover, there is need to consider provision of basic social and healthcare needs such as adequate sleeping space, beddings, water and sanitary services, food and cooking facilities, and ensuring that nurses and midwives conduct regular visits to the mothers staying in the MWHs.

## Background

Globally, around 287 000 women die annually from preventable pregnancy and childbirth-related complications [1, 2]. Almost all (99 %) of the maternal deaths occur in developing countries and more than 50 % occur in sub-Saharan Africa [2, 3]. Zambia is one of the sub-Saharan African countries with a high maternal mortality ratio (MMR) at 398 deaths per 100 000 live births [4]. Most of the maternal deaths and morbidity have been shown to be associated with behavioural risk factors such as low or non utilisation of skilled birth attendance. Skilled birth attendance is considered to be the most important intervention for ensuring optimal maternal and newborn health outcomes [5]. Many maternal and perinatal deaths could be prevented if all women delivered their babies in facilities with adequate resources and staffing that are providing a high quality of medical care [6].

The proportion of women who receive skilled birth attendance is still low in Zambia [4]. Many women (53 %) still give birth at home and most (62 %) do not receive assistance from skilled birth attendants. However, studies have shown that most women who give birth at home express willingness to give birth at the clinic [7–9]. For example, a study by Stekelenburg et al [9] showed that, although most pregnant women (94 %) indicated they would prefer to give birth in a health centre, only 54 % actually did [9].

Rural-urban disparities have been reported in the utilisation of skilled birth attendance services. For example, the United Nations (UN) report [10] showed that, in sub Sub-Saharan Africa, less than half (50 %) of the women in rural areas received skilled attendance at birth compared to over 80 % in urban areas. In Zambia, only 30 % of the women in rural areas are attended to by a skilled provider compared with 80 % of the births in urban women [4]. Several reasons have been reported for the low utilisation of skilled birth attendance services in the rural areas. For example, studies conducted in Zambia [6–9, 11] showed that, in addition to limited access to healthcare facilities due to physical and logistical barriers such as long distances to health facilities and high transportation costs, poor quality of services due to low midwifery staffing levels and a lack of medical equipment for emergence obstetric care, are important reasons preventing pregnant women in rural areas from accessing skilled birth attendance. Further, Sialubanje et al [7, 8] showed that nurses’ disrespectful attitude towards pregnant women, pregnant women’s negative attitude towards healthcare services due to the low quality of services women receive at the clinic, social norms regarding childbirth and indirect costs of buying baby requirements or food while staying in a health care facility are important reasons for pregnant women in rural areas to refrain from accessing skilled birth attendance. Mitigating these barriers could improve the utilisation of skilled birth attendance services [7, 8, 12].

To overcome physical and logistical barriers such as long distances and high transportation costs to healthcare facilities faced by women living in rural areas, maternity waiting homes (MWHs) have been established in many developing countries, including Zambia [13–15]. The World Health Organisation (WHO) has defined MWHs as “residential facilities, located near a qualified medical establishment, where women living far from the healthcare facility and those with high-risk pregnancies can wait for their delivery and be transferred to a nearby medical facility shortly before delivery, or earlier, should complications arise” [15].

Studies investigating the effectiveness of the MWHs have reported positive results. For example, studies from Ethiopia [16], Zimbabwe [17], Liberia [18] and Eritrea [19] as well as a WHO report [15] showed that MWHs improved pregnant women’s access to healthcare facilities, led to an increase in the number of women receiving skilled birth attendance, and reduced maternal mortality in the area and improved maternal and new born health outcomes among women who stayed in the MWHs during the last period of their pregnancy. The report [15] also showed that MWHs were essential in the management of women with high-risk pregnancies. Moreover, a study conducted in rural Zambia [20] comparing women using MWHs and those who did not use them showed that, although women using MWHs had higher maternal risk factors (83 %) compared to those who did not use MWHs (53 %), there were no differences in maternal outcomes between the two groups after delivery, suggesting that MWHs were effective in reducing maternal mortality.

Qualitative studies exploring women’s perceptions towards MWHs show that most women had a positive attitude towards MWHs and expressed willingness to stay in them while waiting for labour.

Regarding women’s perceptions about utilisations of MWHs, qualitative studies show that women’s views differ. For example, in their study conducted in Kalabo district, Zambia, Stekelenburg et al [9] reported that most respondents (97 %) expressed willingness to stay in a MWH if it were available. Similarly, a study evaluating a community trial on MWHs in Liberia [18] showed that traditional midwives participating in the study believed that MWHs provided a safe environment for pregnant women preparing for delivery, allowed them to stay close to the healthcare facilities and helped them rest before giving birth. The study also showed that, compared to the communities without MWHs, those with MWHs experienced a significant increase in the number of births from baseline to post-intervention. In contrast to these positive views, a study from Ghana [21] reported low utilisation of MWHs due to various factors including the cost associated with staying in a MWH, the hardship of staying away from home and the absence of health personnel in healthcare facilities [21, 22].

Little research on women’s perceptions towards MWHs has been conducted. Study findings from other countries may not be applicable to the rural Zambian context. Women’s experiences and opinions regarding utilisation of the MWHs may differ from one geographical, socio-cultural and economic context to another. The purpose of this study, therefore, is to explore women’s experiences and beliefs concerning utilisation of MWHs in rural Zambia. Insight is needed into women’s experiences and beliefs to provide starting points for the design of public health interventions that focus on enhancing access to and utilisation of skilled birth attendance services in rural Zambia, by promoting the use of MWHs.

## Methods

### Study design

The study was qualitative in design and used in-depth interviews (IDIs) to provide a detailed understanding of the women’s experiences and beliefs concerning utilisation of MWHs in Kalomo district. The Tropical Diseases Research Centre Ethics Review Committee and the Ministry of Health Research and Ethics Committee in Zambia granted ethical approval.

### Study setting

The study was conducted in Kalomo district, located 360km south of the capital Lusaka, and covering a total surface area of 15 000 km^2^. It has an estimated population of 275, 779 [23] with an annual growth rate of 4.4 %. Most of the population (92 %) live in rural areas with subsistence farming and cattle rearing being the major economic activities. The district is one of the poorest in the country, with more than 70 % of its population living on less than a dollar per day [24]. Administratively, the district is divided into three constituencies, four chiefdoms and twenty political wards. The health system in the district comprises two hospitals, thirty-four health centres and several health posts. Furthermore, only 52 % of the health care facilities have access to reliable electricity [25] The district is one of the rural districts in the country with low maternal healthcare service utilisation rates, where less than 30 % of the women receive assistance from a skilled birth attendant in a health facility, compared with 80 % of the births in urban women [4, 7, 8, 25]. The main players in the maternal health programmes are the Ministry of Health, missionaries, non-governmental organisations, community leaders and various community-based health agents, including traditional birth attendants.

### Study population and sampling techniques

The study participants were selected from the women of reproductive age (aged between 15 and 45 years) who had given birth within one year prior to the study and were visiting the local health centre for their children’s routine under five clinics. To be eligible to participate in the interview, women must have had resided in the area for more than six months; those who had lived there for less than six months were excluded because the investigators thought these women would not have had enough local experience on utilisation of MWHs in the area. In addition, women aged below 15 and above 45 years were excluded from participation.

Selection of study participants was done using a purposeful homogeneous sampling technique. This technique was used in order to select respondents with similar experience regarding utilisation of MWHs and childbirth services, while, at the same time, allowing for recruitment of respondents with different characteristics in terms of their age, number of children, marital status, and education level, which helped provide insight into the similarities and differences in their experiences [26, 27].

To begin with, all the ten health centres with a MWH in the district were identified and included in the research with the help of the district managers at the District Medical Office. In addition, five (5) out of a total twenty five health centres without a MWH were also purposefully selected and included in the study.

A month prior to the interview, the principal investigator contacted respective health centre in-charges to inform them about the study. Due to logistical challenges, it was not possible to hold meetings with respective health centre in-charges. Instead, they were contacted by phone and the purpose and objective of the study were discussed in detail. The health centre in-charges were then asked to inform the mothers attending the under five clinics about the study and to explain its purpose and objectives−, that is, the study aimed to gain insight into their experience and knowledge about MWHs in their areas, how the decisions for pregnant to use the service were made and what they thought were the main factors affecting utilisation of the service. This information was shared by health centre in-charges during the health promotion sessions conducted by nurses and midwives during each under five clinic visit, and involved all the women attending the under five clinics on a particular day. Women who were willing to participate in the study were advised on the interview date and were asked to return to the clinic for the interview on an agreed upon date. The date for the interview was set by the health centre in-charge and then communicated to the research team through the principal investigator.

### Data collection

The IDIs were conducted from the second week of March, 2014 to the end of May, 2014 and lasted for ten weeks. The research team travelled to the health centre on the day of the interview. To ensure privacy and confidentiality, each IDI was conducted in a quiet place, outside health centre premises, normally under a tree for shade and lasted between 30 and 50 min. The IDIs were conducted in Tonga, the local language in the area. Before each IDI, written consent was obtained from each participant by requesting them to read and sign the consent form, which was translated into the local language. Research assistants read the consent form aloud for those who could not read.

After obtaining consent, research assistants requested each respondent to complete a short demographic questionnaire which included questions such as the respondent’s age, number of children, marital status, level of education, occupation, level of income, estimated walking time to the clinic, place of delivery for the youngest child, history of complications during the previous delivery, and use of a MWH. The last question was only applicable for the respondents located at a health care facility with a MWH. After completing the questionnaire, the interviews were conducted. Each IDI was facilitated by two trained research assistants using a semi-structured interview guide which was translated into Tonga. One research assistant conducted the interview, while the second one recorded using a digital voice recorder. The principal investigator attended interviews at random to ensure the data collection protocol was consistently followed by the research team members.

A total of 32 IDIs were conducted in 9 health centres, 22 of whom were interviewed in 7 health centres with a MWH, and 10 were interviewed in 2 health care facilities without a MWH, although 10 health centres with MWHs and 5 health centres without MWHs were initially identified to be included in the study. After 15 interviews involving respondents from five health centres with MWHs, and 10 interviews from health centres without MWHs, data saturation was achieved; that is, no more substantial information was obtained. At this point, the research team decided to stop the interviews and, thus, leave out the remaining selected health centres. Rather, they decided to only conduct the interviews in the two mission-owned health centres with MWHs in the district. The rationale for this decision was to obtain extra insight into the study from these respondents because, compared to the MWHs in the other health facilities in the district, MWHs in the mission facilities were of better quality and provided better social services such as a larger sleeping space, mattresses, beds and blankets. In addition, the facilities had better cooking facilities and sanitary conditions with piped water. Seven (7) extra respondents were interviewed from these two health centres, giving a total of 32 respondents. The age of the respondents ranged between 17 and 44 years old.

### Research instrument

A semi-structured interview guide was developed that had three pre-determined themes. The first theme focused on women’s perspectives and experience regarding MWHs and its role to improve facility-based skilled birth attendance, and included questions on women’s experience regarding utilisation of MWHs. For example, what they thought about MWHs; whether they had stayed in a MWH before or not; how they felt about their stay in a MWH; what they thought about accessibility to MWHs in their area; whether they would you use it if they were pregnant again and why; what they thought about whether mothers’ shelters were important in helping women deliver at a health centre or not; and if so to explain why and how. The second theme was on the decision-making process regarding utilisation of MWHs and included questions about how the decision is made and who makes it when women want to go to the MWHs. The third theme focused on the important factors which affect women’s actual utilisation of MWHs.

Since there were two different settings (with or without MWHs present) in which the interviews took place, two interview guides were developed reflecting these different settings. The overall themes were the same for both interview guides, however some questions were different. For example, at the health care facility with a MWH, women were asked if and why they did or did not go to stay at the MWH in the last period of their pregnancy. If they did stay in the MWH, their perspectives were explored. At the health care facility without a MWH, women were asked to share their view on MWHs. Furthermore, the women were asked if they would use the MWH if available at the health care facility and why.

### Data analysis

Demographic information was entered into the excel sheet and transferred into IBM SPSS Statistics 21 for processing. Descriptive statistics and frequencies were used to summarise the demographics of the respondents and respective percentages were computed (see Table 1 below).

**Table 1:** Background characteristics of the respondents

Variable	*n* (%)	Mean (SD)
*Mean age in years*		26.8 (7.5)
*Marital status*		
Single	4 (12.5 %)	
*Married*	27 (84.4 %)	
*Divorced*	1 (3.1 %)	
*Number of Children*		3.1 (1.9)
*Level of education*		
Lower primary	1 (3.1 %)	
*Upper primary*	13 (40.6 %)	
*Junior secondary*	14 (43.8 %)	
*Senior secondary*	4 (12.5 %)	
*Occupation*		
*Housewife*	3 (9.4 %)	
*Unemployed*	3 (9.4 %)	
*Farmer*	22 (68.8 %)	
*Self-employed*	3 (9.4 %)	
*Formal employment*	1 (3.1 %)	
*Mean income per month in Zambian kwacha*		183.6 (265.2)
*Walking time to the clinic in minutes*		100.8 (58.1)
*Place of delivery for the youngest child*		
Home	4 (12.5 %)	
*Clinic*	19 (59.4 %)	
*Hospital*	9 (28.1 %)	
*Use of MWHs (only for the 22 respondents from clinics with MWHs)*		
*Yes*	6 (27.3 %)	
*No*	16 (72.7 %)	
*Number of children for those who used MWHs*		3.2
*Number of children for those who did not used MWHs*		2.8
*History of complications during labour*		
Yes	6 (18.8 %)	
No	26 (81.2 %)	
*Health Centres where IDIs were conducted*		
*Health Centres with MWHs*		
*Dimbwe*	4 respondents	
Kanchele	4 respondent	
Mubanga	3 respondent	
Mukwela	2 respondents	
Siachitema	2 respondent	
Namwianga Mission clinic	3 respondents	
Zimba Mission Hospital	4 respondents	
Health Centres without MWHs		
Choonga	4 respondents	
Mawaya	6 respondents	

The voice recordings from the interviews were transcribed and translated into English by the research assistants. To check for accuracy, a few transcripts (20 %) were back − translated into Tonga. Members of the research team then compared the Tonga and English versions for differences and similarities while listening to the original voice recording. After verification of accuracy in translation, each transcript was then thoroughly read by one research assistant while the other one was listening to the corresponding voice recording. Each translated transcript was compared with the hand-written field notes that the research assistants had prepared during the interviews. After proof-reading and making corrections, the transcripts were saved on a password-protected computer. The word documents were then exported into Nvivo 10 MAC for processing. The exported data were then coded and the categories and key sub-themes were identified. In order to make it easy to compare the perspectives of women from the facilities with a MWH and those from health care facilities without a MWH, the data from the two groups of respondents was coded separately. Data analysis was based on the three predetermined themes. An inductive approach was used to derive the sub-themes from the main themes by content-analysing and grouping all the similar statements made with respect to particular themes. Several sub-themes emerged from the data analysis; all sub-themes are described below in the respective sections for the main research themes.

## Results

### Demographics

Table 1 summarizes the demographic characteristics of the 32 respondents included in the study. The mean age was 26.8 years old and the majority (84.4 %) of the respondents were married, and had an average of 3 children. Most of the respondents (68.8 %) were farmers and about two in five (38.7 %) had an income of less than 100 ZMW per month. The estimated walking time to the clinic from the place of the respondents’ residence was one hour and 40 min and the majority (87.5 %) had delivered their youngest child at the health care facility. Of the 32 respondents, 22 were interviewed at a health care facility with a MWH and 10 were interviewed at a health care facility without a MWH. Only 2 out of 22 (9.1 %) of the respondents interviewed at a health care facility with a MWH had a home delivery compared to 2 out of 10 (20 %) that were interviewed at a health care facility without a MWH. Of the 22 respondents interviewed at a health care facility with a MWH, 6 (27.3 %) utilized a MWH and 16 (72.7 %) did not. Out of the 32 respondents interviewed, 6 (18.75 %) experienced complications during labour.

### Theme 1: perspective on maternity waiting homes

The first theme focused on women’s perspectives regarding MWHs as well as accessibility and utilisation of MWHs.

All respondents mentioned that MWHs were important since they helped pregnant women to overcome the problem of having to travel long distances to healthcare facilities. They explained that during the last month of their pregnancy, pregnant women could go and stay in the MWHs and wait for their labour near the health care facility. Respondents, especially those from healthcare facilities without MWHs, added that, with a MWH at the health care facility, women could decide themselves when to leave home to go and stay there instead of having to travel to the clinic when they are already in established labour. They stated that women could either walk from home to the MWHs when they still had the strength to do so or they could use private transport. Respondents mentioned that women who resided far from the health centres experienced delays in reaching the health care facility and that MWHs were especially important for these women. It was also mentioned that MWHs were especially convenient for the women who experienced labour and delivery at night. They explained that MWHs were important for the pregnant women who lived far from the health care facility as it was extremely difficult for them to find transport during the night. They explained that while staying at the MWH, women had immediate access to health care and felt protected against labour complications. Moreover, respondents explained that women were happy that they were able to rest in the MWH before they went into labour. When labour started, it was easy for them to access facility based delivery.

*“It’s a good idea. You can come here if you don’t have transport at your place. You may walk when you still have strength, then you overcome the distance, you stay here waiting for the right time to come, rather than being at home until your time comes and walking a long distance while something is paining” (20-year-old respondent)*

In contrast, most respondents who lived close to the health centres with MWHs believed that they did not need the MWHs because their place was near to the health care facility. Furthermore, most multigravida older respondents preferred to wait at home as they believed they would recognise the labour in time to go to the healthcare facility. They explained that it was only necessary to go to the MWHs if the woman had complications during pregnancy. The young respondents explained that they didn’t have enough knowledge on the MWHs as they had just experienced their first pregnancy. Therefore, they just decided to stay at home and only went to the clinic when they were in established labour.

When asked whether most women had access to the MWHs, all 22 respondents from the health care facilities with a MWH explained that it was easy for women to go and stay there, as there were no rules or regulations regarding the use of the service. In contrast, the 10 women from the health care facilities without MWHs argued that it was hard for them to reach the centres which had MWHs.

Regarding their experience while staying in the MWHs, the 6 respondents who had used the MWHs and most of the older women from the health centres with MWHs complained that women felt abandoned by the healthcare staff as nurses did not check on them and that it was a waste of time staying at the MWHs as it was possible for one to reside there for weeks without being attended to by nurses or midwives. Moreover, the 13 out of the 22 (60 %) respondents who lived close to health care facilities with a MWH but had not used the service were concerned with pregnant women’s inactivity when staying in the MWHs waiting for labour. They argued that it was not good for pregnant women to stay in the MWH because the nurses advised them to rest while staying there. Most older women stated that they preferred to have their labour start earlier and felt that staying active would assist pregnant women have their labour start early. Therefore, respondents explained that some women preferred to stay at home and keep working until the onset of their labour.

When asked whether they would use the MWHs if they were available, all 10 respondents from the health centres without MWHs reported that they would like to see MWHs provided at their health care facility, and that most women would utilise it. They explained that that most women would stop worrying about transport if MWHs were available at their health care facility.

*“If the clinic had the maternal waiting home, the mother shelter here, most pregnant women would come here and stay to wait for her time” (44-year-old respondent)*

When asked whether most women used MWHs where they were available, all the respondents from both the facilities with MWHs and those without reported that most women did not utilise the service. They mentioned that most women did not go to stay in the MWHs because they delayed making the decision to leave home. They explained that although they had planned to stay in the MWH, some women, especially the older ones with many children, went into labour while they were still at home due to lack of transport to take them to the MWHs. Respondents explained that especially young women delayed utilising the MWHs because they had difficulties in estimating the right time to go to the MWHs.

*“I wanted to stay there but the problem was that it was my first pregnancy so I didn’t know when labour would start. So I am planning to come there next time” (24-year-old respondent)*

Regarding the reasons for utilising the MWHs all reported long distances and lack of transport from home to the health care facility as the main reason for utilising the MWHs. Additionally, fear of complications was another important reason for utilising the MWHs. They explained that, often, they were advised by the nurses during antenatal care (ANC) visits to stay in the MWHs. Women who attended ANC at a clinic without a MWH were advised to go and wait for delivery at the district hospital or at a health centre with a MWH.

*“I had some complications; I had some problems so I was told to stay at the clinic before time of delivery” (25-year-old respondent)*

#### Theme 2: decision making process and barriers to utilising a maternity waiting home

The second theme focused on the decision-making process regarding whether or not to utilise MWHs as well as the factors that influence the decision-making process.

Asked about who makes the decision for pregnant women to use the service, most young respondents with few (that is one or two) children from both the health care facilities with MWHs and those without mentioned that the husband is the one who decides whether or not the woman should go and stay at the MWH during the last months of pregnancy. They explained that although most women discussed with their husbands the importance of their stay at the MWHs and often persuaded them to allow their wives to use the MWHs, the final decision whether the woman should use the MWH or not was made by the husband. In addition, young respondents mentioned that the women’s mother and mothers-in law were also involved in the decision-making process. Furthermore, all respondents from both the health care facilities with MWHs and those without also mentioned that nurses at the clinic play an important role in the decision-making process as they often advise women during ANC visits to come and stay at the MWH, especially when there were indications of complications. However, most older respondents mentioned that they made the decision alone and that they did not receive help from anyone in the decision- making process.

*“The husband is the one who decides” (23-year-old respondent)*

*“When we are in the month of 8, the nurses at the clinic tell us to come and wait for our time here, yes” (18-year-old respondent)*

Regarding the factors affecting the decision-making process, respondents who had used MWHs and those who had experienced complications during previous pregnancies mentioned the risk of complications as the major reason to utilise MWHs. Young respondents with no experience with childbirth indicated that fear of complications was the major reason for using MWHs. They explained that as the husband made the final decision, he considered the advice from the nurses at the clinic. They indicated that sometimes during ANC women were told that their baby was not in the right position in the womb, which could cause problems during delivery. Additionally, respondents explained that some women, especially the young ones, were advised by nurses that they would not be able to push the baby out and needed medical help with this process. They also explained that some women, especially the old ones with many children were advised to go and deliver at the clinic because they might bleed a lot after giving birth. If they stay in the MWH before giving birth, women who experience these kinds of complications receive immediate medical help, as they do not experience delays in reaching the clinic.

Asked about which group of women were more at risk of developing complications, respondents mentioned that all women were at risk of developing complications. They explained that especially young women who had no experience with childbirth were at greater risk of developing complications such as prolonged or obstructed labour than older ones with many children. Furthermore, older women who had complications during their previous pregnancies and deliveries were believed to be at a higher risk of developing complications in future pregnancies. Moreover, respondents explained that women (regardless of their age) who were told by nurses during ANC visits that they had pregnancy complications such as the baby not lying well in the uterus, having high blood pressure, etc, were also believed to be more at risk of developing complications during labour. Respondents explained that, compared to the older women with many children, young mothers were more likely to anticipate complications because they were scared of labour complications and believed that they had no experience with childbirth. They explained that many multigravida older respondents believed that they had enough experience with childbirth and that they knew themselves quite well, and that they would know whether they would develop complications or not.

*“Complications, that’s what they look at. Maybe when you deliver at home and you bleed a lot, they won’t help you. But here at the hospital they can control the bleeding. So that is what they consider before deciding with the husband” (23-year-old respondent)*

Another important factor that the families considered during the decision making process is distance. Most respondents interviewed from both the health care facilities with a MWH and those without explained that walking from home to the clinic while in labour pain was difficult when living far from a health centre. Thus, it might be safer to stay at the MWHs before delivering. Additionally, respondents mentioned that availability of transport also plays an important role as the couple decides on whether the woman should go and stay in the MWH or not.

*“The distance, it’s difficult for the woman to walk when she feels the labour pains from home coming here. It’s better for her to come here and stay” (28-year-old respondent)*

Regarding challenges in the decision making process, more than half (18 out of 32) of the respondents interviewed from both the health care facility with a MWH and those without indicated that the lack of a family member to take care of the children and another one to accompany the woman to the MWH and help her while staying there usually made it difficult for the pregnant woman to leave home to go and stay at the clinic. They explained that many women had young children who could not stay by themselves while their mother was away at the MWH awaiting labour. Respondents explained that often, the husband was not able to stay at home to take care of the children due to various commitments, including working in the field.

Another factor that was considered by the wife and husband as they made the decision was the availability of funds to buy the requirements for the baby and mother to use during labour at the clinic. Respondents mentioned that during ANC, nurses advised pregnant women to prepare for childbirth, and that as they went to give birth at the clinic they should carry baby clothes and requirements for the mother such as a wrapper and cleaning materials like bleach. Respondents explained that the husband was expected to find the money for the baby and mother requirements at the clinic. However, respondents explained that most families did not have enough money to buy these items and husbands who failed to provide these requirements refused to allow their wives to go and stay at the MWHs.

Moreover, when making the final decision, husbands considered the availability of people to work in the field. Most respondents who had not used MWHs interviewed at a health centre with MWHs mentioned that some women had not used MWHs because their husbands refused to allow them to leave home due to difficulties of having someone to work in the field, especially during harvest time.

*“There is a challenge of how, who should take care of the children” (36-year-old respondent)*

*“There is no problem, unless this time when we are harvesting, but we have to ask from the husband and say I want to go to the clinic. Husbands will then give us permission” (18-year-old respondent)*

#### Theme 3: factors affecting staying in maternity waiting homes

Respondents were asked if they faced challenges regarding the utilisation of MWHs. Most respondents from both the health care facilities with MWHs and those without mentioned that pregnant women faced many challenges when using the MWHs. Respondents who had used MWHs complained that many MWHs had no beds or mattresses and pregnant women had to carry their own beddings from home. They mentioned that those who failed to carry their own beddings and mattresses had to sleep on the floor. Respondents explained that this was a huge challenge for pregnant women as they had to walk long distances and could not carry beddings on their heads. Similarly, respondents from the health care facilities without a MWHs stressed the need for pregnant women to be comfortable during their stay at the MWHs and that beds and mattresses should be made available.

*“As for now we just sleep on the floor. There are no mattresses unless you bring them from home” (35-year-old respondent)*

*“If they put beds and mattresses it could help women to be delivering at the hospital” (17-year-old respondent)*

Furthermore, respondents who had used the MWHs and most respondents from the healthcare facilities with MWHs stated that the available MWHs had limited space for sleeping. They explained that some MWHs were very crowded because pregnant women came with an accompanying relative. They explained that sometimes women had to sleep outside because of the lack of space.

*“There is no much space in the shelter. Pregnant women need space as they stay at the mother’ shelter. Because of not having enough space women have problems” (25-year-old respondent, IDI30)*

In contrast, all the 7 respondents from the two mission health facilities were happy with the quality of MWHs and the services provided there. They mentioned that the MWHs in the two health facilities had enough space for women and their accompanying relatives and that they had enough beds and mattresses for pregnant women to use. They also indicated that pregnant women staying in these MWHs were provided with blankets by the healthcare staff.

Another important challenge was the lack of food for pregnant women when staying in the MWHs. Respondents who had used MWHs and those from the health care facilities with MWHs mentioned that food was not provided to the pregnant women who stayed in the MWHs. They stated that women had to carry their own food from home. Respondents explained that it was hard for pregnant women to take their own food to the MWHs because they had limited food supplies which were not even enough to share with the other family members remaining at home. Additionally, respondents who had used MWHs indicated that it was usually unknown how long women would stay at the MWHs, and this made it difficult to estimate how much food they needed to take with them. They explained that often, the food ran out before women gave birth. In order to get extra food, women had to travel back home. They explained that while walking home, women risked giving birth on the way.

*“The issue of carrying their own food is a problem. You find that she just has a small amount of food then she has to share it with the family, with children and husband. So it’s a challenge” (41-year-old respondent)*

*“It’s difficult for a mother if the food finishes. She has to go back home. Some women delivered on the way when they went back home to get food” (32-year-old respondent, IDI29)*

Another important problem was lack of water at the MWHs. Most respondents from health care facilities with MWHs stated that most MWHs had no water and women had to walk long distances to get it. They explained that, although in most cases, the accompanying relatives would draw water for the pregnant women, but when the pregnant woman was alone, she had to walk the distance herself. In contrast, the respondents from the two mission health facilities were happy with the quality of the water supply, cooking facilities, and sanitary conditions. They indicated that MWHs in these facilities had running water and pregnant women did not have to walk long distances to look for water as the case was in the other health facilities.

*“As for me, I can’t stay there for one simple reason; there is no water here. It is far where we get water from. They have to improve on the issue of water…” (35-year-old respondent)*

Furthermore, respondents who had used MWHs and those interviewed at a health care facility with MWHs mentioned that the sanitation was poor in the MWHs and needed improvement. They mentioned that some MWHs had no toilets and bathrooms.

In addition, respondents who had used MWHs explained that nurses and midwives did not visit the mothers in the MWHs. They explained that some mothers stayed for a long time in the MWHs without the nurses or midwives checking on them to ensure whether they needed help or not. Respondents explained that the non availability of nurses put most women at risk of complications even if they stayed at the MWHs. They stated that it is important that nurses and midwives make regular visits to the women staying in the MWHs so that those who need care are identified and assisted on time. On the contrary, the 7 respondents from the mission facilities said that most women from these facilities were happy with the way nurses treated them when they used the MWHs. They indicated that nurses usually visited the pregnant women staying in the MWHs and asked those who had problems to go and see the nurse or midwife at the clinic. Moreover, during these visits, the nurses identified the women who had no resources to buy the mother baby requirements and assisted them by providing them with these requirements from the clinic.

*“If nurses are there, there is no problem; even if some of the things are missing there is no problem, just nurses are important” (24-year old respondent)*

## Discussion

The aim of this study was to explore women’s experiences and beliefs concerning utilisation of maternity waiting homes in Kalomo, Zambia. Our findings show that that most women appreciate the important role MWHs play in improving access to skilled birth attendance and improving maternal health outcomes. However several individual, family and health system-related factors prevent utilisation of these services.

Consistent with previous studies which highlighted the importance of MWHs in improving access to facility-based skilled birth attendance [14–22], our findings suggest that most women in rural Zambia have a positive attitude towards this service. Their positive attitude seems to be based on their beliefs and their outcome expectations, i.e. what they would gain from the use of the service. For example, respondents believed that MWHs were an important means to overcome physical barriers such as the long distance to the health care facilities. Furthermore, respondents believed that MWHs were an important means to improve access to skilled and facility-based birth attendance. Moreover, they saw them as able to prevent complications during labour and delivery since pregnant women could easily get assistance from nurses and midwives at the clinic. Moreover, respondents perceived social gains from the use of MWHs. They believed that staying in the MWHs provided pregnant women with an opportunity to rest from the strenuous field work which characterised their livelihood in the rural areas. Staying away from strenuous exercise and resting in the MWHs as they approached their labour was seen as important in improving labour and delivery outcomes for both the mother and the baby. Moreover, these beliefs seem to be based on the respondents’ past experience and information provided by nurses during ANC at the clinic. Thus, MWHs were seen to have many important beneficial effects, not only to improve access to skilled birth attendance, but also to improve mother and birth health outcomes.

This finding is important since it is in line with the original idea of the MWHs which started out as an intervention to improve maternal health outcomes for the women with high-risk pregnancies, but later included pregnant women who had limited access to facility-based skilled birth attendance [15]. Our findings are also in line with a study from Liberia [18] which showed the importance of MWHs in improving access to skilled maternity care. Thus, public health programmes focusing on improving access to skilled birth attendance could benefit from intervention promoting increased availability of MWHs.

Interestingly, although our findings emphasise a positive role MWHs can play in improving access to facility-based skilled birth attendance services in rural areas, the results also show that most women who had access to a MWH did not utilise them because of various factors such as women’s dependence on their husbands for decision-making, non-availability of funds to buy the requirements for the baby and mother to use during labour at the clinic, concerns about a relative to remain at home and take care of the children and concerns about the poor state of the MWHs. This finding is also important as it in line with studies from other developing countries, for example, Ghana [21], which reported low utilisation of MWHs. Further, this finding highlights the complexity of women’ health seeking behaviour and the need for public health interventions to not only focus on the target populations’ attitude and intention, but also to ensure that health promotion interventions target factors that might make it difficult to enact the intended behaviour. Indeed, many studies investigating the implementation of various health behaviours ranging from exercise to breast cancer screening [28–30] have all reported the challenge of “intention-behaviour gap”. For example, a study by Stekelenburg et al in Zambia [14] reported that although the majority of the women (94 %) showed high intentions to use MWHs only half of them (54 %) actually did. Similarly, one of our recent studies [8] which focused on predictors of maternal healthcare service utilisation in rural Zambia showed that most women who had high intentions to give birth in a health facility under skilled birth attendance actually ended up giving birth at home. These findings highlight the need for health promotion interventions to consider providing the requisite skills and resources in order to enable people to implement their intended behaviour.

Regarding the decision to go and stay at the MWHs our findings shed light not only on the decision-making process, but also on an important interplay of factors that determine the pregnant women’s decision to use the MWHs. According to the women that were interviewed, they receive adequate information from the nurses about the existence and the importance of the MWHs. This information is in turn shared with the husband or with parents when the husband is not available. Interestingly, despite discussing the issue of childbirth with her husband, our findings suggest that the pregnant woman does not make the final decision. Rather, the women indicated the husbands play the most important and final role in the decision making process and made the final decision. Women’s dependence on their husband for the final decision could have been as a result of the socio-cultural beliefs recognising the husband as the head of the household and their need for socioeconomic support from the husband. From the interviews, it became apparent that the husband was seen as a provider and women looked up to him for the final decision. Moreover, the dependence on the husband and lack of decision-making autonomy was also perceived as a result of respect for the husband who is perceived to be the head of the household, and hence the decision-maker in most matters affecting the family. This finding is in line with other studies from developing countries [31, 32] which have identified the importance of women’s low socioeconomic status and dependence on their husbands as an important factor which negatively affects women’s health seeking behaviour and prevents them from accessing skilled birth services, and which often leads to adverse maternal health outcomes.

Interestingly, our findings show that, although the distance to the clinic and the woman’s risk of developing complications are the major factors which are considered by the husband as he makes the final decision, our results also suggest that the husband’s final decision is not based solely on the available information from the nurses and midwives. Rather, our results suggest that several other factors such as the availability of funds to buy the requirements for the baby and mother to use during labour at the clinic, the availability of a relative to remain at home and take care of the children, and the availability of people to work in the field when the woman is away, are major influences on the final decision on whether the woman should leave home or not. Our findings suggest that husbands who fail to raise adequate resources to provide for their wives either delay making the decision or stop their wives from leaving home to go and stay at the MWH. Thus, although women discuss child preparedness and matters relating to child birth, the final decision is made by the husband. His decision is based not on the woman’s risk to develop labour and childbirth-related complications and her access to skilled care in case of such complications, but mainly on the availability of resources at the family level. These findings thus clearly show the complexity of the decision-making process regarding women’s utilisation of skilled birth attendance services. The findings are also in line with a study by Thaddeus and Main [13] as well as studies from Ghana and Bangladesh [21, 32] that reported the importance of individual, family and community level factors in delaying or preventing access to maternal healthcare services in developing countries. Thus, our findings highlight the importance of the individual level factors, the immediate family members such as the husband, mother and other relatives, and the community in which the woman lives as important targets for intervention [33]. Moreover, these findings suggest that partner involvement and engaging the community in which the woman lives is an important way to reduce the burden of work on the pregnant women in their late stages of pregnancy to enable them use MWHs.

Another striking finding from our study is how women’s perception of the availability and quality of the basic social and healthcare services provided in the MWHs influences their decision whether to use the service or not. Although dependence on their husbands to be allowed to use MWHs is an important factor limiting pregnant women’s utilisation of MWHs, the results also suggest that before leaving home, women take into consideration other important factors. Among these are the availability and quality of sleeping space, beds and mattresses, water and sanitary facilities, food and the cooking facilities in the MWHs. Our findings suggest that many MWHs did not have these basic facilities and that where they were available, they were either inadequate or not in good quality. These findings are important as they highlight the importance of basic social needs such as shelter, water and food as determinants of skilled birth care utilisation. Before women are expected to use the available service, healthcare systems need to ensure that basic services are provided and basic needs are met [7]. Further, these findings suggest the gains to be made for public health interventions (that is, improved access to MWHs and skilled birth attendance) that focus on providing such needs in the healthcare facilities.

Women also consider their medical safety when staying in the MWHs. For example, most respondents were concerned about the fact that nurses and midwives never visited the pregnant women when they stayed in the MWHs to assess and monitor their conditions. This finding is important as it shows that although the original aim of the MWHs was to increase access to skilled birth attendance; this service may actually not be meeting this aim since women still feel they have no access to skilled care despite staying close to the clinic. This finding is also in line with various studies from Zambia [7–9] which have highlighted the importance of the availability of skilled birth attendants in health facilities in order to increase access to skilled birth attendance as well as improve labour and childbirth outcomes.

Potential limitations of our study should be noted. First, like all qualitative studies, our study findings may not be generalisable to other areas with different socio-cultural, economic and geographical contexts. Furthermore, these findings are only based on the experiences of the few women who accepted to participate in the IDIs; since the recruitment of the respondents was done at the clinic during the routine children’s under five clinic at the health centre, we do not have information on the differences between the women who accepted to participate in the interviews and those who did not. Moreover, although the district has one of the lowest maternal healthcare service utilisation rates [4, 7, 8, 25], most of the respondents interviewed had given birth at the clinic and at the hospital. The reason for this selection was to have women who had experience with MWHs and clinic delivery. However, the selected respondents’ experiences may not be representative of the views of the other women in the community, especially those who had given birth at home. Moreover, interpretation of the findings may have been influenced by the researchers’ individual judgment and experience. Unfortunately, we could not conduct focus group discussions to compare and confirm the findings due to logistical challenges. This could have affected the validity of the findings.

Despite these limitations, we believe our study has provided important insights into the role of MWHs to improve access to skilled facility-based skilled birth attendance. As far as we know, this is the first qualitative study conducted on the subject in rural Zambia.

## Conclusion

In conclusion, our findings suggest that MWHs could be a useful intervention in improving access to, and utilisation of facility-based skilled birth attendance services. Maternity waiting homes are an important means to mitigate long distances to health facilities and to enable women to have access to life-saving interventions during labour and childbirth. MWHs can also assist women to take rest from the laborious field work, which predisposes them to various pregnancy and childbirth related complications [34]. However, currently, these potential benefits are not being realised in rural Zambia due to the various challenges including individual, family, community and health system-related factors which women face when seeking to use the service. These factors include lack of decision-making autonomy and dependence on husbands, prevalent gender inequalities, low socioeconomic status and socio-cultural norms, and concerns about a relative to remain at home and take care of the children, as well as concerns about the poor state and lack of basic social and healthcare needs in the MWHs − such as adequate sleeping space, beddings, water and sanitary services, food and cooking facilities − as well as failure by nurses and midwives to visit the mothers staying in the MWHs to ensure their medical safety all prevent women from using MWHs.

These findings suggest important targets for interventions. For example, our findings suggest a need for an integrated community intervention focusing on supporting families to reduce the burden of work on the pregnant women during the late stages of pregnancy. Moreover, interventions need to focus on recognising and improving the low social status of women and provide them with skills and resources to ensure decision-making autonomy with regard to childbirth. Interventions should focus on promoting partner involvement by providing husbands with knowledge and skills to support their pregnant women in their utilisation of MWHs and facility delivery services. Moreover, although, public health interventions should focus on increasing the number of MWHs, there is also an urgent need to consider the provision of basic needs such as adequate sleeping space, beddings, water and sanitary services, and food and cooking facilities. Further, interventions should focus on increasing the availability of trained and skilled birth attendants such as nurses and midwives to ensure quality of care and medical safety for women both in the MWHs and during labour. Finally, further research is needed to measure and confirm the significance and importance of these findings in determining access to MWHs. Research is also needed to determine whether access to and utilisation of MWHs actually ensure access to skilled birth attendance and improved mother and newborn health outcomes. Findings from those studies can be the basis for advocacy for a public health policy on MWHs in developing countries, which is currently non-existent.
